# Biomethane Conversion of Hemicellulose: Biomethane Production, Kinetic Analysis, Substance Conversion, and Microbial Community Dynamics

**DOI:** 10.3390/bioengineering13030295

**Published:** 2026-03-02

**Authors:** Xiteng Chen, Hairong Yuan, Xiujin Li

**Affiliations:** Department of Environmental Science and Engineering, Beijing University of Chemical Technology, Beijing 100029, China

**Keywords:** anaerobic digestion, hemicellulose, xylan, methane production potential, substance conversion, microbial community

## Abstract

As a key constituent of lignocellulosic biomass, the role of hemicellulose in anaerobic digestion (AD) remains inadequately characterized, particularly regarding its methane potential and degradation process patterns. This study systematically characterized the AD performance of hemicellulose using xylan as a representative substrate. The results showed that xylan achieved a high methane potential of 350–390 mL/g VS and 89.57% biodegradability, exhibiting a shorter lag phase (λ) and higher reaction rate (k) than other biomass fractions. Substantial acetic acid and ethanol accumulated within the first 24 h, while late-stage dissolved organic matter (DOM) shifted toward complex lignin/CRAM-like. The results of microbial dynamics indicated that the collaborative interaction among *Anaerobium*, *Lactobacillus*, and *Clostridium* accelerated xylan transformation. While methanogenesis was predominantly driven by the acetoclastic route (specifically *Methanosarcina*), hydrogenotrophic *Methanobacterium* thrived during temporary pH fluctuations. This work serves as a valuable guide for developing high-performance strategies in industrial lignocellulosic biogas plants.

## 1. Introduction

Anaerobic digestion (AD) has emerged as a mainstream biological approach for valorizing biomass waste into biogas, thus offering carbon-neutral potential for the effective treatment of lignocellulosic waste [1]. Despite the unique advantages of AD in biofuel production and biomass waste valorization, the complexity of biowaste leads to significant fluctuations in digestion performance and biogas yield [2]. Enhancing biomethane conversion efficiency and guaranteeing efficient methane capture—rather than its uncontrolled emission as a potent greenhouse gas—still represent critical challenges for the large-scale application of biogas technology [3]. Lignocellulosic waste, particularly agricultural residues, represents one of the most abundant renewable biomass resources on earth and serves as a promising and cost-effective feedstock for biofuel production [4,5]. However, the inherent structural and biochemical barriers of lignocellulosic biomass severely limit the efficiency of anaerobic digestion. Therefore, extensive studies have focused on substrate pretreatment, nanoparticles and metal ions addition as catalysts, reactor configuration, co-digestion, and microbial engineering for AD [3,6,7]. While pretreatment technologies improve conversion efficiency by disrupting the lignocellulosic structure, a clear understanding of the roles of major lignocellulosic components during anaerobic digestion is critical for guiding the rational application of pretreatment strategies and optimizing process parameters.

The common components of lignocellulosic biomass are lignin, cellulose, and hemicellulose. Previous studies have confirmed that methane is not produced in anaerobic digestion systems where only lignin is used as the substrate [8]. It not only resists degradation but also spatially hinders the interaction between enzymes and substrates. As the primary component, cellulose is a crystalline polymer exhibiting high recalcitrance to microbial degradation [9,10]. Hmaissia and Vaneeckhaute investigated the impact of inoculum sources on the biodegradation performance of sewage sludge and microcrystalline cellulose at both mesophilic (37 °C) and thermophilic (55 °C) temperatures [11]. Song et al. cultivated and acclimated cellulolytic bacterium *Caulobacter* sp. *FMC1* in a liquid medium containing 0.5% cellulose [12]. While extensive research has been devoted to the anaerobic digestion of cellulose and system acclimation using pure cellulose as a substrate, studies focusing on hemicellulose remain comparatively limited. To comprehensively evaluate the AD performance of hemicellulose, it is essential to address three key aspects: the degradation extent of hemicellulose and methane production potential, the transformation patterns of substance throughout the process, and the dynamic succession of the microbial community. As indicated in Table S1, the methane potential of hemicellulose remains poorly defined, as yields are significantly influenced by inoculum activity and system operating conditions. A study by Li et al. identified a maximum biochemical methane potential (BMP) of 355.07 ± 4.23 mL/g VS for xylan [8]; nevertheless, xylan showed negligible methane production in a different fermentation setup [13]. Previous research has primarily focused on final conversion rates and microbial community composition at the endpoint, while largely paying little attention to the temporal dynamics throughout the fermentation process.

Hemicellulose, as a major component of lignocellulosic biomass, is a promising substrate for biomethane production via anaerobic digestion (AD), but its conversion mechanism remains to be systematically clarified. In agricultural residues such as straw, xylan is the predominant hemicellulose, often accounting for over 50% of the total hemicellulose content [14,15,16]. Therefore, this study selected xylan as a representative hemicellulose and conducted biochemical methane potential (BMP) assays at various inoculation ratios to investigate its biogas production performance and methane production potential. Substance conversion during the AD process was evaluated by analyzing soluble chemical oxygen demand (SCOD), xylan content, volatile fatty acids (VFAs) concentration, and the molecular composition of dissolved organic matter (DOM). Furthermore, microbial community structures at different fermentation stages were compared to reveal their successional patterns throughout xylan anaerobic digestion. The findings of this study may provide a theoretical basis and reference for the efficient utilization of hemicellulosic biomass in industrial AD systems.

## 2. Materials and Methods

### 2.1. Substrate and Inoculum

Xylan (X820567, Shanghai Macklin, Shanghai, China) was used as the model pure hemicellulose substrate in this study (Table 1). Inoculum was anaerobic granule sludge from Shandong wastewater treatment. The anaerobic granule sludge was degassed at 35 °C in water bath tank to reduce its methane produced by the endogenesis nutrients.

### 2.2. Biochemical Methane Potential (BMP) Test

The AMPTS II tester (BPC Instruments, Jiaxing, China) was utilized for BMP experiment. The digestion unit simultaneously supported biochemical methane potential test analysis for 15 independent test channels. If the instrument detected that the methane production of a test channel was less than 1% of the total gas production for three consecutive days during the fermentation process, it was recorded as the end of fermentation. The anaerobic digestion unit in this experiment had a reaction volume of 400 mL and was conducted at mesophilic conditions (35 ± 0.5 °C). Before adding the substrate, the carbon-to-nitrogen ratio was adjusted to 25:1 and the initial pH was set at 7.5. The BMP methane potential tester mechanically stirred the mixture at 100 rpm for 5 min every hour. The inoculum to substrate ratio (ISR) was set at 2:1, 3:1, and 4:1 (anaerobic granule sludge: substrate, based on VS). The TS value of anaerobic digestion system was controlled at around 8%. An only inoculum group was set up to subtract the background gas production value of the anaerobic sludge. In addition, three replicates were set for each experimental group. During the anaerobic digestion process, the daily gas production volume and the content of each gas component in the biogas were recorded. The gas production results were converted to standard conditions (273.15 K and 101.325 kPa).

### 2.3. Analytical Methods

#### 2.3.1. Physical and Chemical Properties

The biogas components (H_2_, CH_4_ and CO_2_) were measured using gas chromatography (GC-2014c, Shimadzu, Kyoto, Japan) equipped with a TDX-01 column and a thermal conductivity detector (TCD). High-purity argon (99.999%) was used as the carrier gas with a flow rate of 30 mL/min. Concentrations of each component were quantified via external standard method, and results were expressed as % (*v*/*v*). Total solids (TS), volatile solids (VS), ammonia nitrogen and pH were analyzed following standard methods (APHA 2012) [17]. For the determination of ethanol, volatile fatty acids (VFAs), and soluble chemical oxygen demand (SCOD), they were centrifuged at 9000 rpm for 15 min and filtered through a 0.22 μm membrane. SCOD was assessed using a Lianhua Technology COD Rapid-Test Kit (LH-DE-100, Beijing Lianhua Yongxing Technology Development Co., Ltd., Beijing, China), and UV–visible spectrophotometer (UV2200, Shanghai Sunny Hengping Scientific Instrument Co., Ltd., Shanghai, China). Ethanol and VFAs were analyzed using gas chromatography with high-purity nitrogen as the carrier gas (GC-2014c, Shimadzu, Kyoto, Japan).

Xylan detection was performed using a high-performance liquid chromatography (HPLC1260, Agilent, Memphis, TN, USA) equipped with a refractive index detector (RID). Aminex HPX-87H sugar analysis column (Bio-Rad, Hercules, CA, USA) was employed for separation. The mobile phase consisted of 5 mmol/L sulfuric acid (0.5 mL/min), the column temperature was maintained at 55 °C, and the injection volume was set at 20 μL. The first-order kinetic model was widely used to characterize the hydrolysis kinetics in the AD process of solid particulate materials [18,19]. The consumption of xylan in BMP tests was described by Equation (1). Where S_0_ is the initial concentration of solid organic matter, S_t_ stands for the concentration of xylan during hydrolysis period in this study, and k_deg_ represents the first-order kinetic hydrolysis rate constant (d^−1^).

(1) $$S_{t}=S_{0}\times \exp\left(-k_{deg}\times t\right)$$

In order to verify the changes in DOM components under anaerobic digestion of xylan (model hemicellulose), samples were selected in digestion sequence according to biogas production conditions. Fourier transform ion cyclotron resonance mass spectrometry (FT-ICR-MS) analyses were performed using a SoleriX 15T FT-ICR-MS platform (Bruke, Berlin, Germany), employing an electrospray ionization (ESI) source in negative ion mode. The raw water sample (12 mL) was filtered through a 0.22 μm filter to eliminate particulates and impurities, then acidified by dropwise addition of formic acid to adjust the sample pH to 2. Solid-phase extraction (SPE) of dissolved organic matter (DOM) in the sample was subsequently carried out with an Agilent Bond Elut PPL cartridge (1.0 g, 6 mL). Prior to sample detection, the instrument was calibrated with a 10 mmol/L sodium format solution. After sample analysis, internal standard calibration was performed using soluble organic matter with a known molecular formula, and all mass errors of the detections were less than 1 ppm following calibration. During subsequent data processing, seven categories prevalent in natural DOM were classified based on H/C and O/C ratios [20,21]: Lipids for H/C = 1.5–2.0, O/C = 0–0.3; aliphatic/proteins for H/C = 1.5–2.2, O/C = 0.3–0.67; lignin/carboxylic rich alicyclic molecules (CRAM-like) for H/C = 0.7–1.5, O/C = 0.1–0.67; carbohydrates for H/C = 1.5–2.4, O/C = 0.67–1.2; unsaturated hydrocarbons for H/C = 0.7–1.5, O/C = 0–0.1; aromatic structures for H/C = 0.2–0.7, O/C = 0–0.67; and tannin for H/C = 0.6–1.5, O/C = 0.67–1.

#### 2.3.2. Kinetic Analysis

The Modified Gompertz, Logistic, Cone, and First-order kinetic model equations were used to fit the cumulative biomethane yield data, as shown in Equations (2)–(5). Where B represents the cumulative methane production (mL/g VS), B_0_ is the maximum potential methane production (mL/g VS) and R_max_ is the maximum methane production rate (mL/g VS/d). λ is the lag phase (d), t represents the reaction time (d), k is the rate constant (d^−1^), and e is the mathematical constant approximately equal to 2.7183.

Based on the elemental composition of hemicellulose, the theoretical methane production (TMP) was calculated through the total biochemical reaction equation of anaerobic digestion, as shown in Equations (6) and (7). The experimental methane production (EMP) refers to the cumulative methane production obtained in experimental groups. The biodegradability (BD) of the raw materials was calculated in Equation (8).

(2) $$B=B_{0}\times \exp\left\{-\exp\left[\frac{R_{max}\times e}{B_{0}}\left(\lambda -t\right)+1\right]\right\}$$

(3) $$B=\frac{B_{0}}{1+exp\left[\frac{{4R}_{max}}{B_{0}}\left(\lambda -t\right)+2\right]}$$

(4) $$B=\frac{B_{0}}{1+{\left(kt\right)}^{-n}}$$

(5) $$B=B_{0}\times \left[1-\exp\left(-kt\right)\right]$$

(6) $$C_{n}H_{a}O_{b}N_{c}+\left(n-\frac{a}{4}-\frac{b}{2}+\frac{3c}{4}\right)H_{2}O\to \left(\frac{n}{2}+\frac{a}{8}-\frac{b}{4}-\frac{3c}{8}\right){CH}_{4}+\;\left(\frac{n}{2}-\frac{a}{8}+\frac{b}{4}+\frac{3c}{8}\right){CO}_{2}+c{NH}_{3}$$

(7) $$TMP=\frac{22.4\times 1000\times \left(\frac{n}{2}+\frac{a}{8}-\frac{b}{4}-\frac{3c}{8}\right)}{12n+a+16b+14c}$$

(8) $$BD=\frac{EMP}{TMP}\times 100\%$$

### 2.4. Microbial Community Analysis

Microbial samples from anaerobic digestion digestates at different times of HC2 were collected and immediately stored at −20 °C prior to 16S rRNA gene high-throughput sequencing. Total microbial genomic DNA was extracted using the E.Z.N.A.^®^ soil DNA Kit (Omega Bio-tek, Norcross, GA, USA) according to manufacturer’s instructions. The hypervariable regions V3–V4 of the bacterial 16S rRNA gene were amplified with primer pairs 338F (5′-ACTCCTACGGGAGGCAGCAG-3′) and 806R (5′-GGACTACHVGGGTWTCTAAT-3′). For archaeal community analysis, primer pair 524F10ext and Arch958Rmod were used [22]. All PCR reactions were carried out with 15 µL of Phusion^®^ High-Fidelity PCR Master Mix (New England Biolabs, Ipswich, MA, USA). Sequencing libraries were generated using TruSeq^®^ DNA PCR-Free Sample Preparation Kit (Illumina, San Diego, CA, USA) following manufacturer’s recommendations and index codes were added. The library quality was assessed on the Qubit@ 2.0 Fluorometer (Thermo Scientific, Waltham, MA, USA). At last, the library was sequenced on an Illumina NovaSeq platform and 250 bp paired-end reads were generated.

### 2.5. Statistical Analysis

Data were analyzed using Microsoft Excel 2021, and Origin 2026 was used for data fitting. All data were averaged for the physical and chemical test results. DOM components-related calculations were performed on the website (https://domnetweb.com) for statistical analysis. Correlation heatmap was generated using the ‘linkET’ package (available at: https://github.com/Hy4m/linkET (accessed on 20 February 2026)) in R software (version 4.1.3).

## 3. Results and Discussions

### 3.1. Biomethane Production Performance

#### 3.1.1. Methane Production Potential

**Figure 1:**
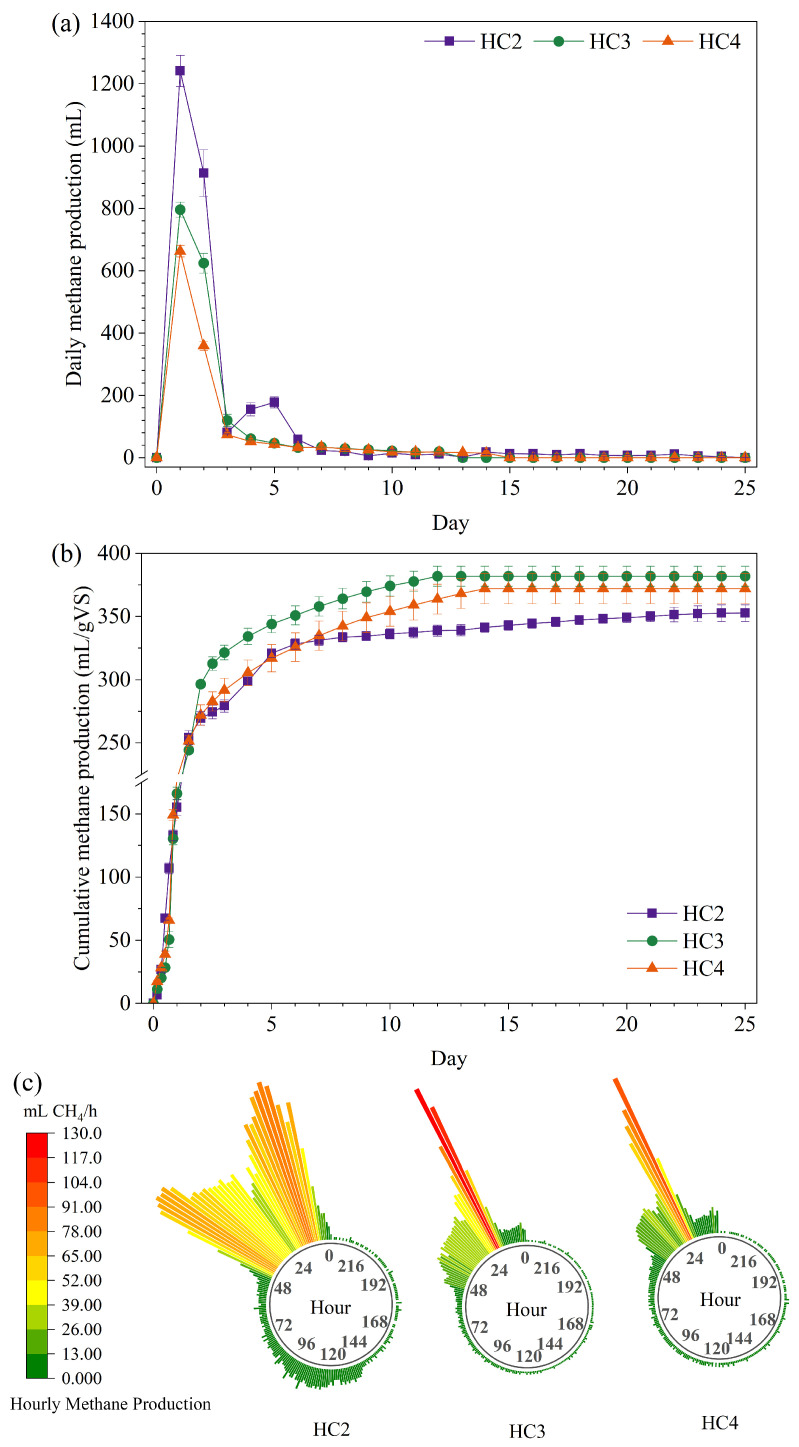
Daily methane production (**a**), cumulative methane production (**b**), and hourly methane production (**c**).

The methane production of xylan (model hemicellulose) at different inoculation ratios was investigated. As shown in Figure 1, the highest cumulative methane production was obtained in the HC3 group (381.91 ± 7.95 mL/g VS). In the HC2 and HC4 test groups, the cumulative methane production was 352.70 ± 6.58 mL/g VS and 372.17 ± 12.00 mL/g VS, respectively. Xylan, as the main component of plant hemicellulose, is one abundant polysaccharide in nature. The methane-generation trend in the experimental group revealed that xylan was rapidly decomposed and utilized by anaerobic microorganisms. Although numerous studies have investigated the anaerobic digestion performance of xylan during its bioconversion to methane, the cumulative methane production reported in these studies was barely higher than that observed in the present work [23,24,25,26]. This discrepancy can be attributed to two factors in previous research: a lower inoculum-to-substrate ratio (ISR) and suboptimal inoculum activity. The cumulative methane productions of xylan were 202.90 ± 3.40 mL/g VS and 211.00 ± 9.00 mL/g VS reported by Li et al. and Hua et al. [23,25]. Nevertheless, the XY group did not produce detectable methane in a prior study [13]. Under high ISR and long HRTs, Li et al. and Saady et al. reported methane yields of xylan were 355.07 ± 4.23 mL/g VS (ISR = 1, HRT = 50 d) and 310.5 ± 3.4 mL/g VS (ISR = 3.4, HRT = 98 d) respectively [8,27]. The inoculum to substrate ratio was 2 to 4 in this study, and this high value assured that the biological system was not overloaded. Properly elevating the inoculation amount could boost biomethane yield, but the highest inoculation ratio did not correspond to the maximum biomethane yield [28,29]. Methane production of HC4 was lower than that of HC3. This may be attributed to competition and substrate consumption among microbial communities. The optimal methane production was obtained in HC3 (ISR = 3, HRT = 25 d). Thus, based on the experimental data, the methane production potential of xylan (model hemicellulose) in this study was approximately 350–390 mL/g VS, and the maximum biodegradation rate reached 89.57%. These values are higher than those reported previously, supporting the reliability of the experimental results (Table S1).

A gas production peak was observed in all three experimental groups during the initial two days of the anaerobic digestion cycle (Figure 1a). Notably, the maximum daily methane production during fermentation appeared on the 1st day, with values of 1241.15 ± 50.08 mL (HC2), 795.98 ± 23.92 mL (HC3) and 662.58 ± 18.93 mL (HC4) for the three groups, respectively. Notably, the methane content was less than 50% (*v*/*v*) before 24 h but rose to 60% (*v*/*v*) at 36 h. The methanogenic stage of XY was predominantly confined to the initial two days of anaerobic fermentation. From the 3rd to the 6th day of anaerobic fermentation, the HC2 group presented a stage of rising daily methane production, showing a significantly higher growth rate than the other experimental groups, with the maximum daily methane production reaching 177.96 ± 17.69 mL. The HC2 group received a higher xylan addition than the HC3 and HC4 groups, and high concentration substrate biodegradation generated more volatile fatty acids and methanogenic precursors. However, the low initial microbial abundance in the HC2 group resulted in an inevitable lag in gas production. Consistent with this observation, the same trend had been reported in a relevant study. Li et al. reported that two methane production peak periods were identified during the XY fermentation process (ISR = 1), with one at 20–25 days and the other at 30–35 days [23]. Low ISR induced overload of the biological system, leading to compromised functional stability. After the 6th day of anaerobic digestion, daily methane yields gradually decreased, while the cumulative biogas production and cumulative methane production approached stability (Figure S1c,d).

The 1st day of anaerobic fermentation was T50 for all three groups (Table S1). As illustrated in Figure 1c, maximum hourly methane production was detected in all experimental groups during this stage, demonstrating that hemicellulose underwent rapid and complete degradation and subsequent utilization by the anaerobic microbial consortium. T90 of HC2, HC3 and HC4 were 4 d, 4 d and 5d, respectively, and their corresponding T95 were 10 d, 8 d and 10 d. Notably, the highest cumulative methane yield was obtained in HC3, which also had a significantly shorter fermentation duration than the other two groups. Therefore, xylan (model hemicellulose) is capable of rapid and complete transformation and utilization by anaerobic microbial consortia, and its methane production potential has been verified in the present study.

#### 3.1.2. Kinetics Analysis of Methane Yield

Table 2 presents the kinetic parameters obtained from the four models frequently used for the methane production in this study [30,31]. The Modified Gompertz and Logistic models could forecast lag time (λ) and maximum biogas production rate (B_0_) during AD. The Cone and First-order models could determine reaction rate constant (k) in an accurate manner [32]. The R^2^ of each group ranged from 99.02% to 99.41%, reflecting that the Cone model had good curve-fitting performance for the data. The excellent simulation performance of the Cone model had been extensively reported in previous studies [33,34]. The poor fit of the First-order model may be partly attributed to the early-stage inhibition caused by rapid acid accumulation and pH decrease. The fitting results of the cone model indicated the B_0_ value of HC3 was higher than that of HC2. The results under the other three models also indicated that B_0_ was positively correlated with ISR [32], which is attributed to abundant microbial populations and high microbial diversity during organic matter decomposition. In contrast, R_max_, λ, and k failed to adequately simulate this trend, which was inconsistent with the findings of previous studies [29]. This discrepancy was attributed to the significantly higher xylan addition in the low-inoculum-ratio system compared with the high ISR system. Specifically, the elevated organic loading of the xylan substrate promoted its hydrolytic efficiency and utilization efficiency by microorganisms.

Xylan, as a pure hemicellulose substance, had a shorter lag phase for methane production compared with other biomass substrates (e.g., straw and kitchen waste) [34,35]. The First-order model rate constant (k) ranged between 0.55 and 0.64 d^−1^, and the Cone constant rate ranged between 0.83 and 0.93 d^−1^. The reported values for xylan were higher than those reported in the literature (0.08–0.40 d^−1^) [18,36,37,38]. This highlighted the superior performance of the inoculum used in this study, since high kinetic constants were obtained for pure hemicellulose matter without the need for inoculum pretreatment to accelerate AD efficiency, especially for HC3 under mesophilic conditions. As indicated in Table 2, the apparent hydrolysis rate constant (k) predicted by the First-order model for HC3 was higher than the observed values for HC2 and HC4 respectively. Furthermore, it ensured the reliability of the methane production potential of xylan (model hemicellulose) in this study and avoided experimental interference caused by the poor performance of inoculum.

### 3.2. Substance Conversion

#### 3.2.1. Variation of Xylan Content

**Figure 2:**
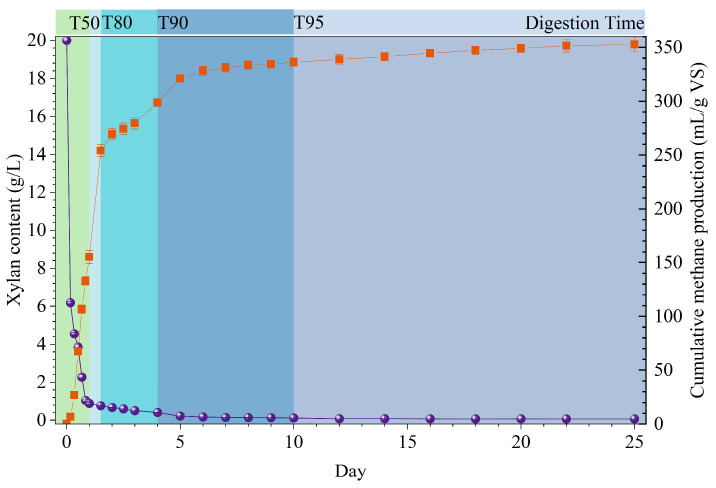
Xylan content and cumulative methane production in HC2. The purple circle line indicates the xylan content, the orange square line indicates the cumulative methane production, and the colored boxes denote fermentation periods in chronological order.

The variation of xylan content during the 25-day anaerobic fermentation, as shown in Figure 2, was positively correlated with methane production. The digestion time (T50) required to reach 50% of the maximum cumulative methane production was 1 day, and the xylan degradation rate was 95.60% at the same time. On the 4th day (T90) and 25th day, the xylan removal rates reached 97.96% and 99.67%, respectively, indicating that xylan (hemicellulose) was a rapidly and completely biodegradable substrate. The First-order kinetic model was widely used to characterize the hydrolysis kinetics in the AD process of solid particulate materials. The k_deg_ value for xylan at T90 was 0.97 d^−1^. The apparent hydrolysis rate constant (k) of HC2 using the First-order kinetic equation was 0.62 d^−1^ in Table 2. Notably, the k value of the methane yield was significantly lower than the hydrolysis rates of xylan (k_deg_). This result deviated from previous research, which proposed that hydrolysis was the rate-limiting step in the AD system or that the rate-limiting step only shifted from hydrolysis to the acetogenic stage [19,39]. As shown in Figure S3, this may suggest that methanogenesis becomes transiently rate-limiting in the initial stage of AD, as evidenced by the accumulation of acetic acid. The rapid accumulation of acetic acid indicated efficient hydrolysis and acidogenesis, whereas methanogenic archaea could not timely convert the acetic acid into methane. The subsequent acid accumulation and pH drop further suppressed methanogenic activity, making methanogenesis the transiently rate-limiting step for methane production. This situation is more likely attributed to the inherent properties of xylan rather than organic loading, since methanogenesis still did not proceed immediately at the beginning of the experiment even though the organic loading rates in HC3 and HC4 were lower than that in HC2.

#### 3.2.2. Variation in pH Value, SCOD, Ethanol and VFAs

Temporal variations in pH and SCOD during AD were illustrated in Figure S2. During the 1st day in HC2, the pH value declined sharply from 7.5 to 6.2. Meanwhile, the xylan content and SCOD concentration also decreased rapidly. The pH value increased at the 24th hour and recovered to above 7.0 by the 36th hour, with the methane content in biogas reaching over 60%. Eventually, the pH continued to rise and stabilized at 7.8–8.0. The SCOD concentration reached its peak at the 4th hour and subsequently decreased gradually, reflecting excellent solubility of xylan, and strong capabilities of anaerobic microorganisms to transform and utilize this substrate.

As key intermediates in the AD process of converting substrates to methane, VFAs had a significant impact on biogas production depending on composition structure [40]. VFAs concentration increased rapidly at the 4th hour, indicating intensive hydrolysis and acidogenesis of xylan. And acetic acid was the predominant VFA throughout the fermentation process (accounting for 99.93%), which suggested that acetic-type fermentation was favored under the AD conditions [8]. Ma et al. performed batch fermentation experiments using potato peels and food waste, observing that acetic acid was the dominant product [41]. Additionally, butyric acid and ethanol concentrations increased with higher carbohydrate content in the substrate. During 8–24 h of anaerobic fermentation, ethanol concentration increased significantly with the decline in pH. Under nearly neutral pH conditions, the volatile fatty acids (VFAs) produced predominantly by glucose fermentation were acetate and butyrate acid, while ethanol was the fermentation product under more acidic conditions [42]. On the other hand, excess hemicellulosic substrate (xylan) resulted in over-threshold monosaccharides from hydrolysis (substrate inhibition) due to abundant available substrate for microbes. Butyric acid accumulated significantly from the 12th hour to the 3rd day of anaerobic digestion (Figure S3). It had also been reported that butyric acid was produced in relatively high concentrations [8,13]. This might be attributed to the operational performance recovery of the acidification system. The recovery of the system may be mainly due to the gradual consumption of accumulated VFAs by methanogens (rather than ammonia release), which alleviated acid inhibition and restored system stability (Figure S2). During the recovery of pH, butyrate-producing bacteria exhibited higher efficiency in utilizing monosaccharides (glucose and xylose), which could be further utilized by anaerobic microorganisms.

### 3.3. Characteristics of Dissolved Organic Matter

**Figure 3:**
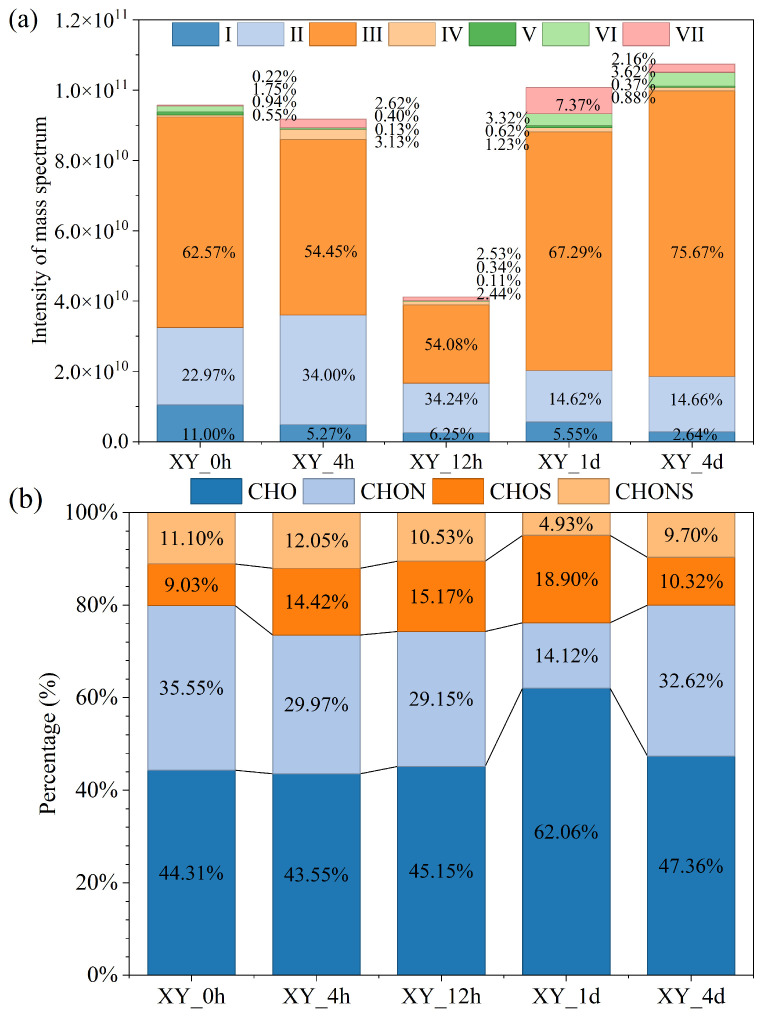
Intensity of mass spectrum of DOM (**a**), and DOM element composition basing on relative intensity (**b**).

Dissolved organic matter (DOM) was reported to be biologically reactive and influencing the operation of the anaerobic system [43]. To elucidate the transformation of substances during the actual hydrolysis process, based on the methane yield, this research studied the changes in the composition of DOM at different fermentation times (0 h, 4 h, 12 h, 1 d (24 h), and 4 d). The assigned formulae were categorized as follows, and as illustrated in the Van Krevelen (VK) diagram (Figure S4): lipids (I), aliphatic/proteins (II), lignin/CRAM-like (III), carbohydrates (IV), unsaturated hydrocarbons (V), aromatic structures (VI), and tannins (VII) [28]. The VK diagram of 0 h (initial anaerobic granular sludge) is shown in Figure S4a. It was observed that lignin/CRAM-like (62.57%), aliphatic/proteins (22.97%), and lipids (11.00%) predominated in the liquid phase of XY_0h (Figure 3a). Previous studies on sludge and other similar substrates are consistent with this study [44,45,46]. Since no lignin was supplemented to the AD system, classes III were predominantly composed of CRAM-like. Results showed that the contents of lignin/CRAM-like of XY_1d (67.29%) and XY_4d (75.67%) were higher compared with XY_4h and XY_12h (54.45% and 54.08%, respectively) in the DOM derived from the xylan AD system. In contrast, the contents of easily degradable biomolecules such as lipids, aliphatic/proteins, and carbohydrates were relatively lower in the XY_1d and XY_4d. At the 4th and 12th hour, the relative peak intensity proportion of classes I and II increased by 5.30% and 6.52%, respectively, which was consistent with the increasing trend of VFA concentrations. As the primary precursors of VFAs, lipids and aliphatic/proteinaceous compounds provided additional intermediates for methanogenesis, and exhibited relatively higher bioavailability [47]. These shifts in molecular composition reflected a transformation from unstable structures to more stable and aromatic ones, enhancing carbon retention and system performance. During the rapid gas production period, the carbon flow was prioritized towards the generation of acetic acid/ethanol, reducing the aromatization diversion and the formation of new inhibitory aromatics. In the later stage of anaerobic digestion, increased lignin/CRAM-like compound content was linked to greater complexity and stability of the DOM structure. As anaerobic digestion proceeded, the amount of DOM in class VII (tannins) increased, with its relative abundance reaching 7.37% in XY_1d. During anaerobic digestion, microorganisms preferentially utilize readily degradable components to produce refractory by-products featuring lower molecular weight, reduced unsaturation, and enhanced aromaticity [48]. As tannins exhibited high unsaturation and aromaticity, an increased proportion of these components reflected a marked enhancement in the biological activity of the xylan-degrading microbial community. The DOM molecules were primarily divided into CHO, CHON, CHOS, and CHONS based on the elemental composition (Figure 3b, Table S3). Accordingly, CHO and CHON groups were two main organic substances in DOM of AD. In XY_1d (24th hour), the CHO fraction rose remarkably due to pH-rebound-activated hemicellulose (xylan) hydrolysis, lagged methanogenic consumption and desorption of bound CHO. The increase in CRAM-like components at the late stage of digestion is most likely derived from microbial metabolites. In addition, the increase in CRAM-like fractions might be attributed to the fact that sufficient carbohydrates provided energy for the transformation and polymerization of aromatic compounds. Coupled with the continuous accumulation of recalcitrant CRAM-like components, the CRAM-like fractions increased accordingly.

### 3.4. Microbial Community Composition and Diversity

**Figure 4:**
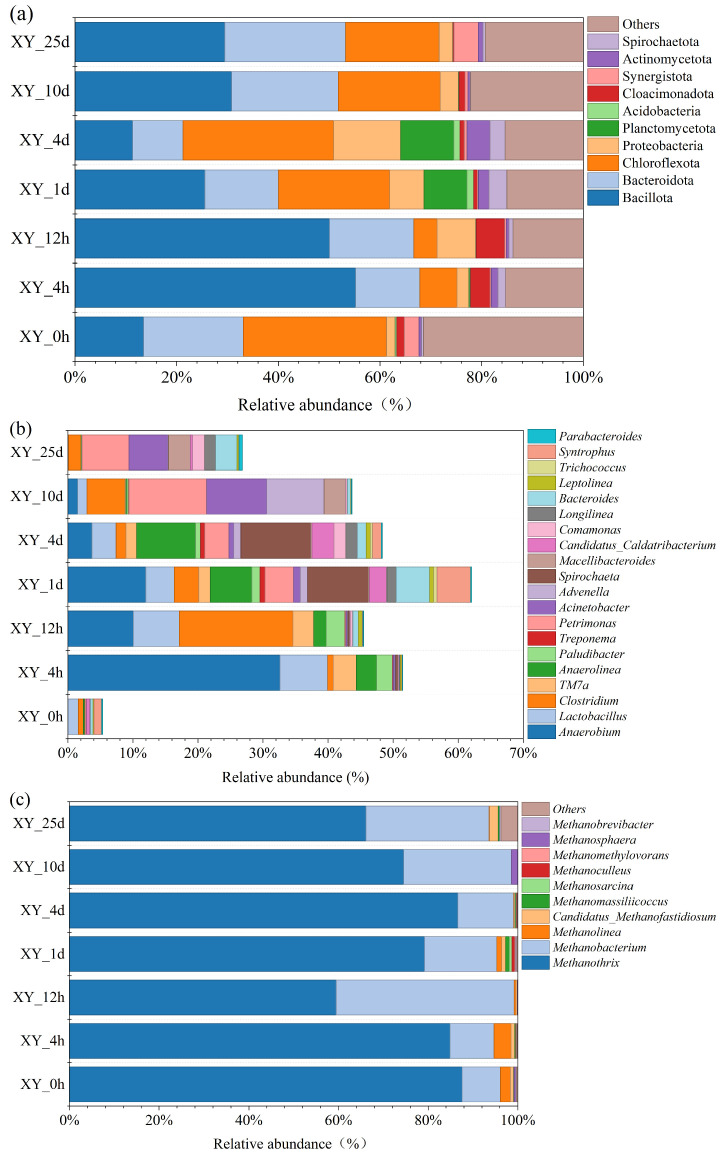
Microbial community composition and diversity: relative abundance of bacteria at the phylum level (**a**), genus level (**b**), and archaea at the genus level (**c**).

For further investigation of the temporal variation in the microbial community during the anaerobic digestion of xylan (hemicellulose), representative samples from HC2 reactor at 0 h, 4 h, 12 h, 1 d (24 h), 4 d, 10 d, and 25 d were selected for microbial community composition and diversity analysis. As shown in Figure 4, Bacillota and Bacteroidota were the dominant bacteria at the phylum level during the AD process (1st day). The relative abundance of Bacillota rapidly rose to 55.20% (XY_4h), and it remained at 50.05% (XY_12h) and 25.55% (XY_1d). Meanwhile, Bacteroidota were 12.61% in XY_4h, rose to 16.58% in XY_12h, and decreased to 14.44% in XY_1d. They constituted the core functional groups for hemicellulose hydrolysis, acidification, and acid production [8,13]. Compared with XY_0h, Chloroflexota (formerly Chloroflexi) decreased from 28.18% to 7.33% in XY_4h and further to 4.59% in XY_12h. After the acidification period ended and the pH value of the system was restored, the relative abundance of Chloroflexota began to increase significantly, reaching 21.87% in XY_1d, 29.61% in XY_4d, 20.03% in XY_10d, and 18.42% in XY_25d. This is due to the high sensitivity of Chloroflexota to abrupt pH changes, as this phylum is adapted to a pH range of 6.5 to 7.0. As shown in Figure 4b, *Anaerolinea* (phylum Chloroflexota) exhibited a relatively high relative abundance in XY_1d and XY_4d, but a low relative abundance in XY_4h and XY_12h.

During the first four days of anaerobic digestion in the HC2 group, the relative abundance of unclassified phyla (Others) declined to approximately 15% from 31.46% at the start-up time (XY_0h). This result suggested that the microbial community rapidly shifted from an initially dispersed composition to a state dominated by core functional taxa with xylan-utilizing capacity. Notably, the relative abundance of *Anaerobium* reached 32.60% in XY_4h, with *Anaerobium_acetethylicum* as the dominant strain (Figure S5). *Anaerobium_acetethylicum* (family Lachnospiraceae, genus *Anaerobium*) is a strictly anaerobic functional bacterium that efficiently ferments diverse carbohydrates including xylose, yielding ethanol, acetic acid, hydrogen and other key intermediate metabolites, and has been reported to be widely distributed in xylose-based anaerobic digestion systems [8,13]. In XY_12h, the relative abundance of *Lactobacillus* and *Clostridium* increased (7.07%, and 17.42% respectively). *Anaerobium* accounted for 10.07% in the reactor. Although its relative abundance slightly decreased, it remained at a relatively high level in the AD reactor. This microbial abundance pattern directly reflects niche differentiation and functional complementarity among the three genera, which are shaped by their inherent acid tolerance, metabolic characteristics, and substrate utilization spectra. *Lactobacillus* maintains stable xylose fermentation and acid production under acidic stress, which is the primary driver of its increased relative abundance. However, its metabolic restriction to efficiently utilizing only small-molecule monosaccharides limits its population expansion. In contrast, *Clostridium* displays moderate acid tolerance, and its endospore-forming ability allows it to effectively withstand acidic conditions. More significantly, it possesses a broader substrate spectrum that includes both Xylo oligosaccharides and monosaccharides. Its capacity to generate mixed volatile fatty acids meets the diverse metabolic needs of the anaerobic system, thereby expanding its ecological niche and establishing it as the dominant acid-producing genus with a higher relative abundance.

At the initial stage of digestion (XY_0h), the archaeal community was primarily composed of *Methanothrix* and *Methanobacterium*, with relative abundances of 87.58% and 8.53%, respectively. During the process of AD, the primary dominant archaeal genus in the reactor was still *Methanothrix*. The percent of community abundance of *Methanobacterium* increased significantly under low pH conditions (XY_12h). Furthermore, its proportion in the later stages of anaerobic digestion was also notably higher than that in XY_0. *Methanobacterium* primarily produces methane using hydrogen and carbon dioxide, with some strains also capable of utilizing formate as a carbon source [45]. It can adapt to diverse environmental conditions, including temperature, pH and salinity [44]. However, the initial acidification during xylan digestion results in an excessively high hydrogen partial pressure in the system. Consequently, the enrichment of hydrogenotrophic methanogens (*Methanobacterium*) is essential to alleviate the hydrogen partial pressure. Once hydrogen partial pressure is sufficiently reduced, acetotrophic methanogens begin to generate methane via acetic acid decomposition. Thus, despite the high percent of community abundance of *Methanobacterium*, methane production via the hydrogenotrophic pathway remained limited. In the anaerobic digestion of xylan (hemicellulose), methane was mainly produced through the acetoclastic pathway, which was primarily mediated by *Methanothrix* [8].

**Figure 5:**
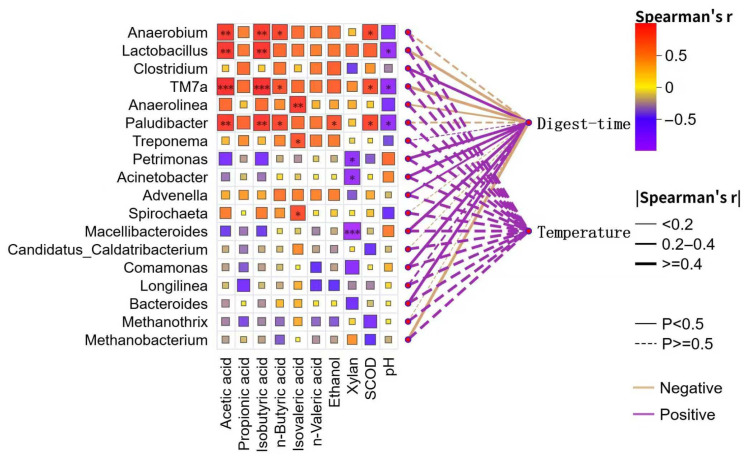
Correlation heatmap and network analysis between key microbial genera and process parameters. “*”, “**” and “***” indicate significant correlations at *p* < 0.05, *p* < 0.01 and *p* < 0.001, respectively.

Spearman correlation analysis revealed that representative fermentative/acidogenic genera (i.e., *Anaerobium*, *Lactobacillus*, *Candidatus Saccharibacteria* (*TM7a*), and *Paludibacter*) were significantly positively correlated with several volatile fatty acids and SCOD (*p* < 0.01), indicating synchronous accumulation during the hydrolysis–acidification phase (As shown in Figure 5). Methanogenic genera (*Methanothrix*, *Methanobacterium*) were generally weakly negatively correlated with VFAs but positively correlated with pH, consistent with the inhibition of methanogenesis under acidic conditions. Network analysis further indicated enrichment of most fermentative taxa with increasing digestion time, whereas *Anaerobium* and *Lactobacillus* declined at later stages and were more closely associated with xylan, reflecting their activity in early polysaccharide hydrolysis. Overall, fermentative consortia drive increases in VFAs and SCOD, whereas excessive acidification may suppress methanogenesis; these findings highlight the need for the close monitoring and control of pH and VFA profiles during early polysaccharide anaerobic digestion to maintain process stability.

### 3.5. Economic Evaluation

As reported in previous studies, improving the biogas yield efficiency of anaerobic digestion can boost the electricity output of biogas power generation and promote economic benefits [49]. However, process instability can inhibit methanogenesis. This consequent reduction in biogas yield lowers the net present value, increases waste management costs, and significantly undermines overall profitability [50]. This study also confirmed that an increase in methane yield is conducive to improving economic returns as shown in Table 3. Under the Combined Cycle Gas Turbine (power generation efficiency = 60%) with a feed-in tariff of 0.75 CNY/kWh, the economic benefits of this study were significantly higher than those of the other two previously reported studies, by 276.45% and 470.04% respectively.

## 4. Conclusions

This study explored the methane production potential, methane yield kinetic analysis, substance conversion, and microbial community dynamics of hemicellulose represented by xylan in anaerobic digestion (AD) systems. Results showed that the methane production potential of xylan (model hemicellulose) was approximately 350–390 mL/g VS, and the maximum biodegradation value was 89.57%. In addition, xylan was almost completely utilized in the AD system. The kinetic fitting results indicate that xylan exhibits a shorter lag phase (λ) but a higher reaction rate constant (k) during anaerobic digestion compared with other biomass. The high degradability of xylan leads to substantial acetic acid production on the first day of anaerobic digestion, with a certain amount of ethanol generated simultaneously. In the later stage of anaerobic digestion, increased lignin/CRAM-like compound content was linked to greater complexity and stability of the DOM structure. The synergistic relationship among *Anaerobium*, *Lactobacillus*, and *Clostridium* facilitated the conversion of xylan to methane. Biomethane was primarily generated via the acetoclastic pathway dominated by *Methanothrix*, and the relative abundance of *Methanobacterium* increased significantly when the pH of the AD system decreased (XY_12h). Enhancing methane production from xylan is critical to improve the economic feasibility of anaerobic digestion.

## Figures and Tables

**Table 1:** Characteristics of substrate and inoculum.

Characteristics	Anaerobic Granule Sludge	Xylan
TS (%) ^a^	8.74 ± 0.01	95.83 ± 0.87
VS (%) ^a^	5.96 ± 0.08	95.79 ± 0.86
NH_3_-N (mg/L)	784 ± 2.36	N.A.
pH	7.96 ± 0.01	N.A.
Lignin (%) ^b^	0.68 ± 0.03	0.00
Cellulose (%) ^b^	1.64 ± 0.09	0.00
Hemicellulose (%) ^b^	2.58 ± 0.13	100.00
C (%) ^b^	38.86 ± 0.24	42.08 ± 0.05
H (%) ^b^	5.48 ± 0.27	6.60 ± 0.03
N (%) ^b^	8.74 ± 0.07	0.01 ± 0.00
O (%) ^b^	42.76 ± 0.38	50.98 ± 0.02
Grain size (mesh)	N.A.	100

^a^ based on wet weight, ^b^ based on dry weight, N.A. means Not Applicable.

**Table 2:** Kinetic parameters of methane yield.

Model	ISR	B_0_ (mL/g VS)	R_max_ (mL/g VS/d)	λ (d)	R^2^ (%)	k (d^−1^)
Modified Gompertz model	HC2	338.10	169.14	0.10	98.10	N.A.
	HC3	373.66	167.01	0.13	98.33	N.A.
	HC4	357.19	166.46	0.13	96.85	N.A.
Logistics model	HC2	335.82	180.39	0.19	97.05	N.A.
	HC3	371.28	172.94	0.20	97.52	N.A.
	HC4	354.66	176.26	0.22	95.67	N.A.
Cone model	HC2	346.85	N.A.	N.A.	99.41	0.93
	HC3	373.58	N.A.	N.A.	99.28	0.85
	HC4	369.59	N.A.	N.A.	99.02	0.83
First-order kinetic model	HC2	343.76	N.A.	N.A.	98.35	0.62
	HC3	374.72	N.A.	N.A.	98.67	0.64
	HC4	364.76	N.A.	N.A.	97.33	0.55

N.A. means Not Applicable.

**Table 3:** Economic benefits from methane-based power generation under three different treatments.

Substrate	EMP (mL CH_4_/g VS)	Digestion Time (Day)	SMY (mL CH_4_)	Power Generation (kWh)	Economic Benefit (CNY)	References
Xylan	202.90	50	405.80	2.43 × 10^−3^	1.82 × 10^−3^	Li et al. [23]
Xylan	133.90	50	267.80	1.60 × 10^−3^	1.20 × 10^−3^	Ma et al. [24]
Xylan	381.91	25	1527.64	9.13 × 10^−3^	6.85 × 10^−3^	This study

SMY: specific methane yield per unit VS over the 100-day fermentation period.

## Data Availability

The original contributions presented in this study are included in the article/Supplementary Materials. Further inquiries can be directed to the corresponding author.

## Supplementary Materials

The following supporting information can be downloaded at: https://www.mdpi.com/article/10.3390/bioengineering13030295/s1. Figure S1. Anaerobic digestion performance (a) methane content, (b) CO_2_ content; (c) cumulative biogas production, and (d) cumulative methane production. Figure S2. pH value and SCOD concentration during AD process of HC2. Figure S3. Ethanol and VFAs concentration during AD process of HC2. Figure S4. VK diagram of DOM components at different time of anaerobic digestion system of HC2: 0 h (a), 4 h (b), 12 h (c), 1 d (d), 4 d (e). Figure S5. Relative abundance of top five species in different AD groups (a) XY_4h, (b) XY_12h, (c) XY_1d, (d) XY_4d, (e) XY_10d, and (f) XY_25d. Table S1. Biomethane production performance of hemicellulose in literatures. Table S2. Anaerobic digestion performance of this study. Table S3. Molecular characterization of AD digestates during AD process of HC2. Supplementary data related to this article can be found at (https://doi.org/10.5281/zenodo.18798629). References [51,52,53,54] are cited in the supplementary materials.
