# Supplementary material for: Biomethane Conversion of Hemicellulose: Biomethane Production, Kinetic Analysis, Substance Conversion, and Microbial Community Dynamics
Source: Bioengineering (Basel). 2026 Mar 2;13(3):295. doi: 10.3390/bioengineering13030295 (PMC13023624; doi:10.3390/bioengineering13030295)
Supplement: Supplementary file 1 [file bioengineering-13-00295-s001.zip › bioengineering-4174740-supplementary.pdf]

Supplementary Materials

Biomethane Conversion of Hemicellulose: Biomethane Production, Kinetic Analysis, Substance Conversion, and Microbial Community Dynamics

Xiteng Chen, Hairong Yuan and Xiujin Li *
Department of Environmental Science and Engineering, Beijing University of Chemical Technology, Beijing 100029, China
* Correspondence: xjlibuct@163.com

There are five figures and three data tables in this supplementary Materials:

Figure. S1. Anaerobic digestion performance (a) methane content, (b) CO_2 content; (c) cumulative biogas production, and (d) cumulative methane production.

Figure. S2. pH value and SCOD concentration during AD process of HC2.

Figure. S3. Ethanol and VFAs concentration during AD process of HC2.

Figure. S4. VK diagram of DOM components at different time of anaerobic digestion system of HC2: 0 h (a), 4 h (b), 12 h (c), 1 d (d), 4 d (e).

Figure. S5. Relative abundance of top five species in different AD groups (a) XY_4h, (b) XY_12h, (c) XY_1d, (d) XY_4d, (e) XY_10d, and (f) XY_25d.

Table. S1 Biomethane production performance of hemicellulose in literatures.

Table. S2 Anaerobic digestion performance of this study.

Table. S3 Molecular characterization of AD digestates during AD process of HC2.

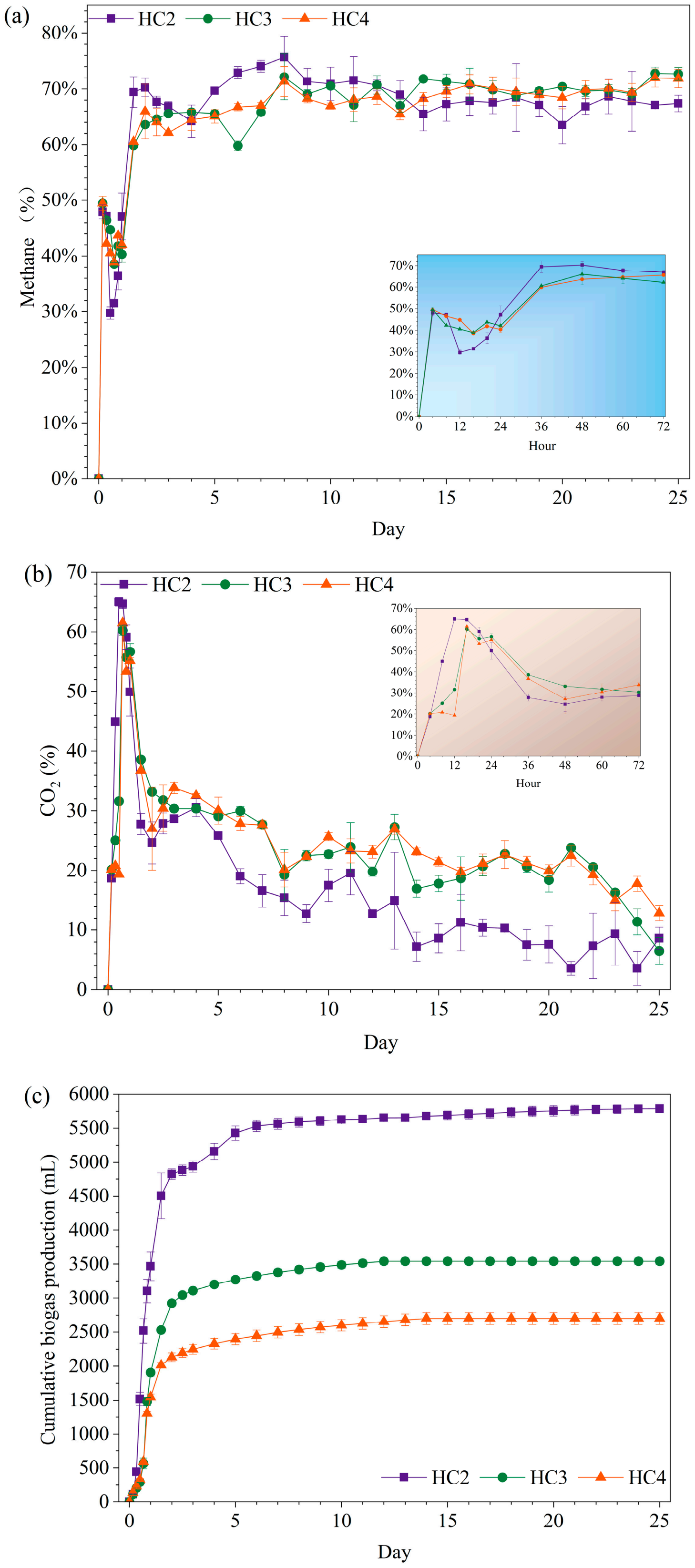
(a)
HC2
HC3
HC4
Methane （%）
Day
Hour
(b)
CO2 (%)
Day
Hour
(c)
Cumulative biogas production (mL)
Day

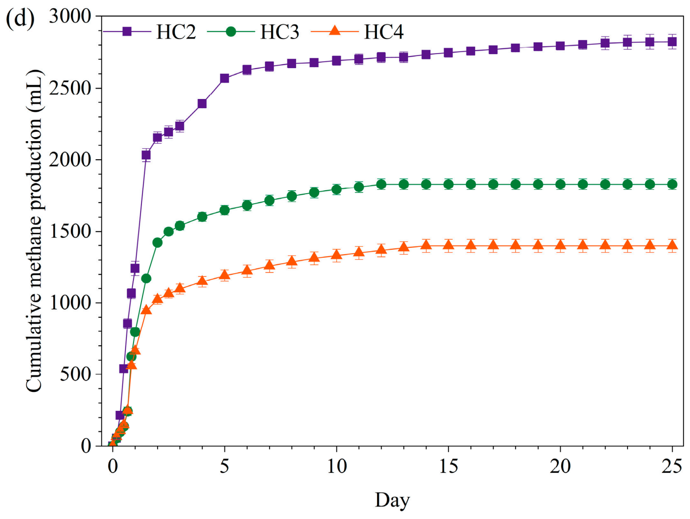

Figure. S1. Anaerobic digestion performance (a) methane content, (b) CO_2 content; (c) cumulative biogas production, and (d) cumulative methane production.

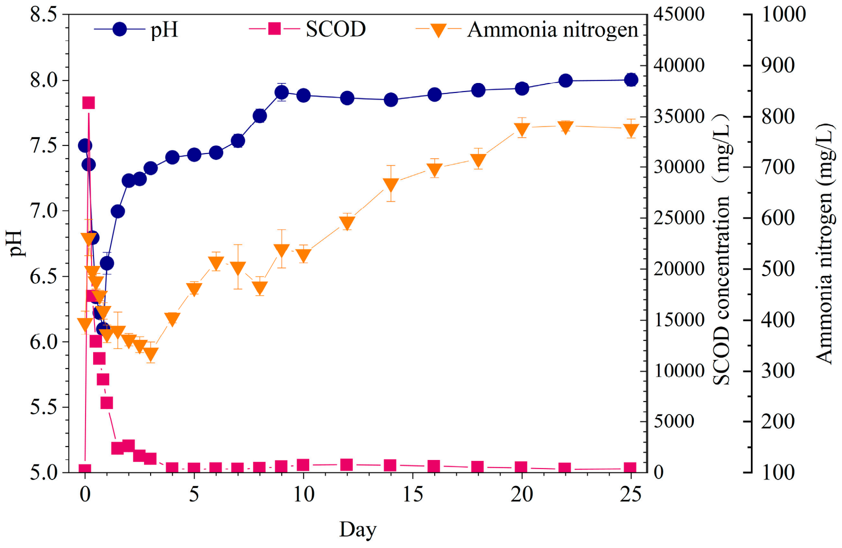

Figure. S2. pH value, Ammonia nitrogen and SCOD concentration during AD process of HC2.

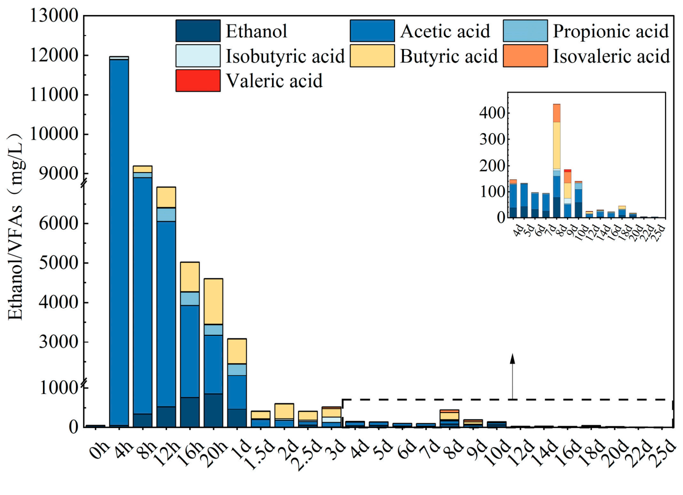

Figure. S3. Ethanol and VFAs concentration during AD process of HC2

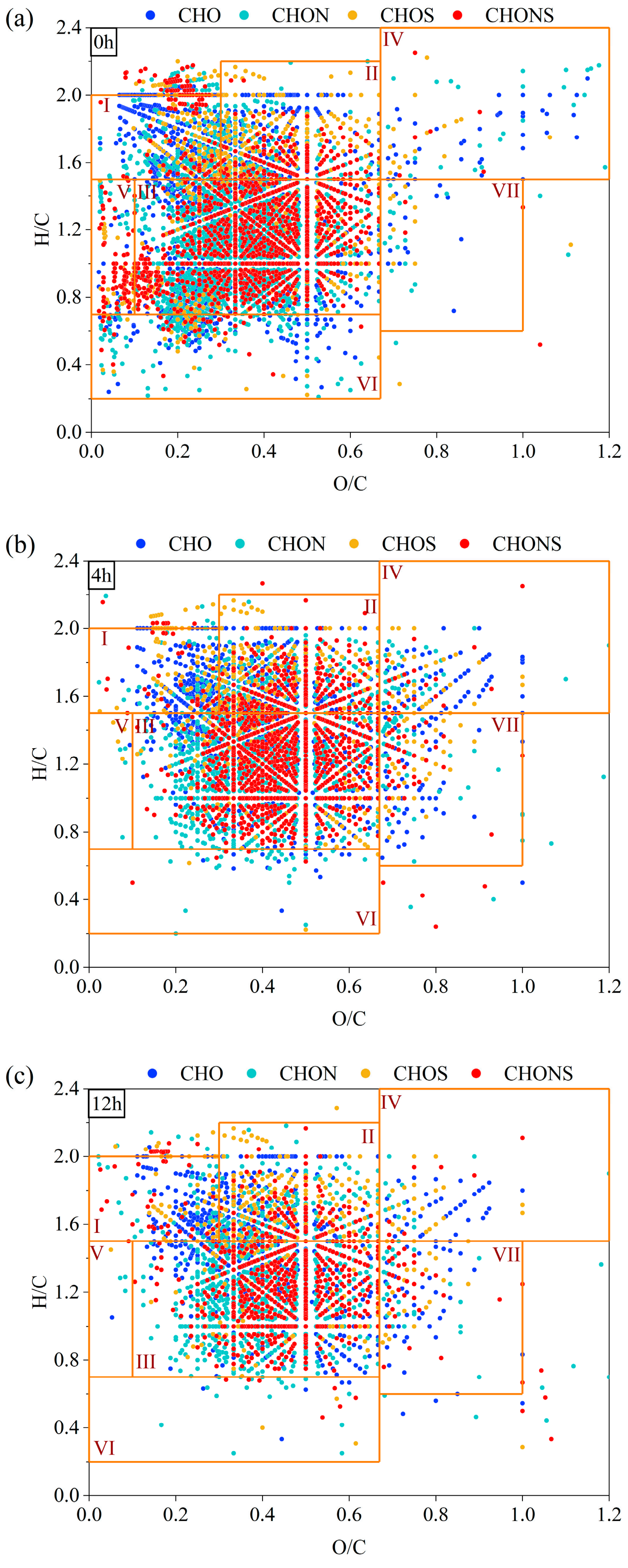

(a)
CHO
CHON
CHOS
CHONS
0h
I
II
III
IV
V
VI
VII
H/C
O/C
(b)
CHO
CHON
CHOS
CHONS
4h
I
II
III
IV
V
VI
VII
H/C
O/C
(c)
CHO
CHON
CHOS
CHONS
12h
I
II
III
IV
V
VI
VII
H/C
O/C

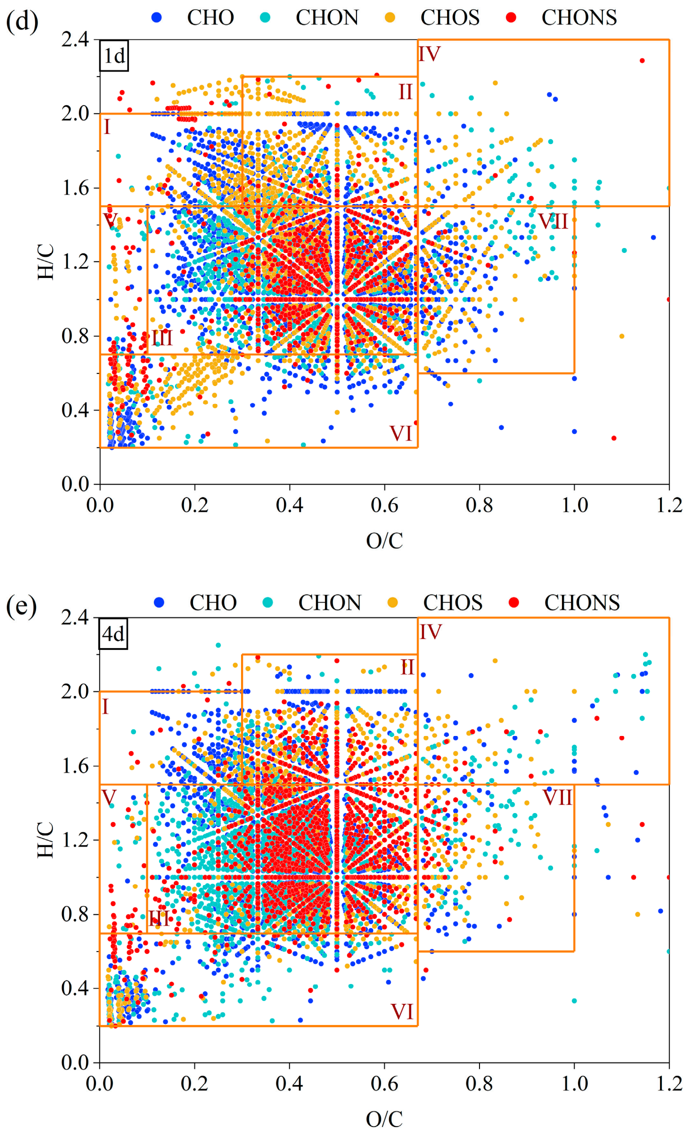

Figure. S4. VK diagram of DOM components at different time of anaerobic digestion system of HC2: 0 h (a), 4 h (b), 12 h (c), 1 d (d), 4 d (e).

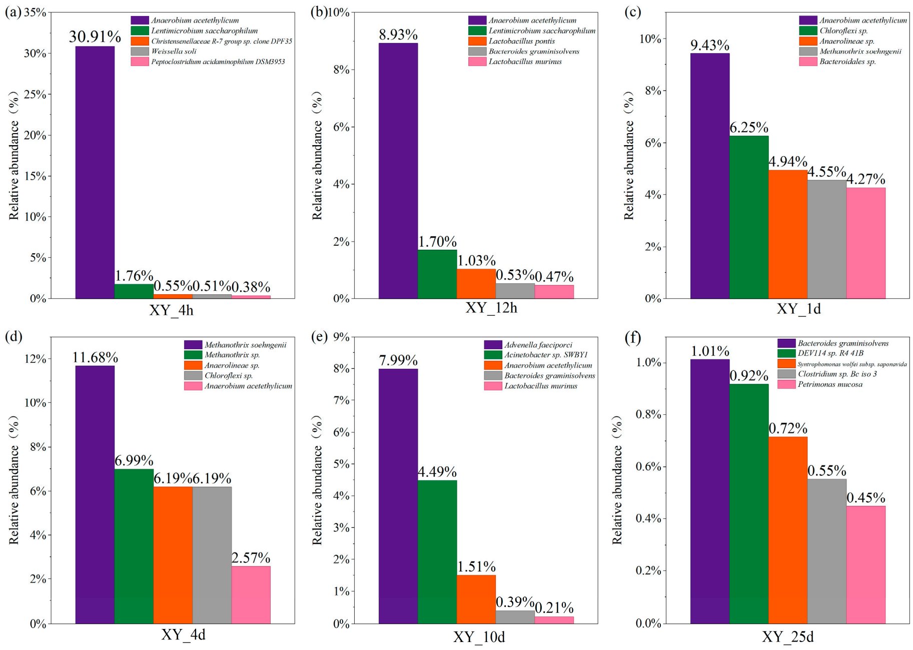

Figure. S5. Relative abundance of top five species in different AD groups (a) XY_4h, (b) XY_12h, (c) XY_1d, (d) XY_4d, (e) XY_10d, and (f) XY_25d.

Table. S1 Biomethane production performance of hemicellulose in literatures

Substrate	Inoculum/Source	ISR (basis)	Methane yield (mL CH_4/g VS)	Biodegradability (%)	Temperature (°C)	HRT (days)	References
Xylan	Anaerobic granule sludge	2:1(VS)	352.70 ± 6.58	82.72	35	25	**This study**
Xylan	Anaerobic granule sludge	3:1(VS)	381.91 ± 7.95	89.57	35	25	
Xylan	Anaerobic granule sludge	4:1(VS)	372.17 ± 12.00	87.28	35	25	
Xylan	Anaerobic sludge from pig manure AD plant	1:1 (VS)	202.90 ± 3.40	49.20 ± 2.20	37	50	Li et al.[1]
Glucomannan	Anaerobic sludge from pig manure AD plant	1:1 (VS)	178.60 ± 5.50	43.60 ± 6.00	37	50	
Arabinogalactan	Anaerobic sludge from pig manure AD plant	1:1 (VS)	223.50 ± 5.00	55.30 ± 5.30	37	50	
Xylan	Anaerobic continuous reactor (pig manure + straw)	1:1 (VS)	133.90	35.00	36	50	Ma et al.[2]
Xylan	Wastewater treatment plant sludge anaerobic digester	1:2 (VS)	211.00 ± 9.00	N.A.	37	N.A.	Hua et al.[3]
Xylans polymers (Arabinose/Xylose= 0.32)	Anaerobic granular sludge (mesophilic sugar factory digester)	2.5:1 (VS)	245.05	N.A.	35	24	Barakat et al.[4]
Xylans polymers (Arabinose/Xylose= 0.10)	Anaerobic granular sludge (mesophilic sugar factory digester)	2.5:1 (VS)	265.76	N.A.	35	24	
Xylans polymers (Arabinose/Xylose= 0.59)	Anaerobic granular sludge (mesophilic sugar factory digester)	2.5:1 (VS)	219.80	N.A.	35	24	
Xylans polymers (Arabinose/Xylose= 0.28)	Anaerobic granular sludge (sugar factory digester)	2.5:1 (VS)	240.00	N.A.	35	24	

Mannose	Mixed anaerobic active sludge (not acclimated to lignocellulosic feedstock)	2:1 (TS)	235.97	63.21	37	90	Li et al.[5]
Galactose	Mixed anaerobic active sludge (not acclimated to lignocellulosic feedstock)	2:1 (TS)	261.99	70.18	37	90	
Xylan	Mixed anaerobic active sludge (not acclimated to lignocellulosic feedstock)	2:1 (TS)	190.34	44.86	37	90	
Glucomannan	Mixed anaerobic active sludge (not acclimated to lignocellulosic feedstock)	2:1 (TS)	193.01	46.53	37	90	
Xylan	Anaerobic sludge from dairy manure AD plant	3:1 (VS)	312.26	N.A.	37	90	Varongchayakul et al.[6]
Xylan	Anaerobic sludge from pig manure AD plant	3:1 (VS)	277.64	N.A.	37	90	
Xylan	Anaerobic sludge from goat manure AD plant	3:1 (VS)	142.07	N.A.	37	90	
Xylan	Anaerobic sludge from Napier grass AD plant	3:1 (VS)	90.14	N.A.	37	90	
Xylan polymers (Arabinose/xylan =0.32:1)	Mesophilic AD reactor (sugar factory)	5:1 (VS)	241.00 ± 3.00	N.A.	35	30	Barakat et al.[7]
Xylan polymers (Arabinose/xylan =0.12:1)	Mesophilic AD reactor (sugar factory)	5:1 (VS)	263.00 ± 4.00	N.A.	35	30	
Xylan	Wastewater treatment plant anaerobic digester	1:1 (TS)	355.07 ± 4.23	N.A.	36	50	Li et al.[8]
Xylan	Wastewater treatment plant CSTR sludge	1:1 (TS)	6.73	N.A.	36	50	Li et al.[9]
Xylan	Sequencing batch AD (dairy manure + wheat straw)	5.6:1 (VS)	310.50 ± 3.40	78.87	20	N.A.	Ahmed et al.[10]

Table. S2 Anaerobic digestion performance of this study

Group	XY addition (g)	CBP (mL/g VS)	EMP (mL/g VS)	TMP (mL/g VS)	BD (%)	T50 (d)	T80 (d)	T90 (d)	T95 (d)	k_{deg} (d^{-1})
HC2	8.35	723.64	352.70	426.40	82.72	1	1.5	4	10	0.97
HC3	5.00	739.37	381.91	426.40	89.57	1	2.0	4	8	0.88
HC4	3.92	719.13	372.17	426.40	87.28	1	1.5	5	10	0.83

*CBP, Cumulative biogas production; EMP, Experimental methane production; TMP, Theoretical methane production; BD, Biodegradability.

Table. S3 Molecular characterization of AD digestates during AD process of HC2

Samples	XY_0h	XY_4h	XY_12h	XY_1d	XY_4d
CHO (%)	44.31%	43.55%	45.15%	62.06%	47.36%
CHON (%)	35.55%	29.97%	29.15%	14.12%	32.62%
CHOS (%)	9.03%	14.42%	15.17%	18.90%	10.32%
CHONS (%)	11.10%	12.05%	10.53%	4.93%	9.70%
Total formulas (n)	7844	5939	3977	7825	8719
MS	387.40	363.43	357.35	408.92	417.25
O/C	0.36	0.73	0.44	0.46	0.44
H/C	1.34	1.16	1.36	1.20	1.14
DBE	7.79	6.91	6.60	9.08	10.33
AI_{mod}	0.23	0.15	0.17	0.27	0.31
NSOC	-0.47	0.44	-0.34	-0.22	-0.13
KMD_{CH2}	0.25	0.24	0.25	0.26	0.25
KMD_{CO2}	-0.20	-0.15	-0.21	-0.21	-0.21
KMD_{H2}	0.00	-0.02	0.00	0.00	0.00

*The values are abundance-weighted. MS, molecular mass; O/C and H/C, average elemental ratios; DBE, double bond equivalent; Al_{mod}, modified aromaticity index; NOSC, nominal oxidation state of carbon; KMD_{CH2}, CH2-based Kendrick mass defect; KMD_{H2O}, H2O-based Kendrick mass defect; KMD_{CO2}, CO2-based Kendrick mass defect (used to identify homologous series associated with alkyl chain variation, hydration/dehydration, and carboxylation/decarboxylation, respectively)[11].

References

[1] Li W W, Khalid H, Zhu Z, et al. Methane production through anaerobic digestion: Participation and digestion characteristics of cellulose, hemicellulose and lignin[J]. Applied Energy, 2018, 226: 1219-1228.

[2] Ma S, Wang H, Li J, et al. Methane production performances of different compositions in lignocellulosic biomass through anaerobic digestion[J]. Energy, 2019, 189.

[3] Hua Y, Song Q, Li L, et al. Non-additive effect: Variation in the effects of the interaction between primitive components in anaerobic digestion[J]. Resources, Conservation and Recycling, 2024, 204: 107507.

[4] Barakat A, Kadimi A, Steyer J P, et al. Impact of xylan structure and lignin–xylan association on methane production from C5-sugars[J]. Biomass and Bioenergy,

2014, 63: 33-45.
[5] Qiumin Li. Effect of the composition of three components of lignocellulose on methane production by anaerobic digestion [D]. Yunnan Normal University, 2024.
[6] Varongchayakul S, Songkasiri W, Chaiprasert P. High potential lignocellulose-degrading microbial seed exploration from various biogas plants for methane production[J]. Renewable Energy, 2024, 231: 120900.
[7] Barakat A, Gaillard C, Steyer J P, et al. Anaerobic Biodegradation of Cellulose-Xylan-Lignin Nanocomposites as Model Assemblies of Lignocellulosic Biomass[J]. Waste and Biomass Valorization, 2014, 5(2): 293-304.
[8] Li P, Cheng C, Guo R, et al. Interactions among the components of artificial biomass during their anaerobic digestion with and without sewage sludge[J]. Energy, 2022, 261: 125130.
[9] Li P, Wang Y, Cheng C, et al. Investigation of interactions among major biomass components during anaerobic digestion under pH-adjustment conditions[J]. Journal of Environmental Chemical Engineering, 2025, 13(2): 115667.
[10] Ahmed A M S, Buezo K A, Saady N M C. Adapting anaerobic consortium to pure and complex lignocellulose substrates at low temperature: kinetics evaluation[J]. International Journal of Recycling of Organic Waste in Agriculture, 2019, 8(1): 99-110.
[11] Hu A, Li L, Huang Y, et al. Photochemical transformation mechanisms of dissolved organic matters (DOM) derived from different bio-stabilization sludge[J]. Environment International, 2022, 169: 107534.
